# High expression of CD52 in adipocytes: a potential therapeutic target for obesity with type 2 diabetes

**DOI:** 10.18632/aging.202714

**Published:** 2021-03-11

**Authors:** Rui Mao, Fan Yang, Yu Zhang, Hongtao Liu, Pengsen Guo, Yanjun Liu, Tongtong Zhang

**Affiliations:** 1 The Center of Gastrointestinal and Minimally Invasive Surgery, The Third People’s Hospital of Chengdu, Affiliated Hospital of Southwest Jiaotong University, Chengdu 610031, China; 2 Emergency Department, Third Clinical Medical College, Peking University, Beijing 100191, China; 3 Medical Research Center, The Third People’s Hospital of Chengdu, The Second Chengdu Hospital Affiliated to Chongqing Medical University, Chengdu 610031, China

**Keywords:** CD52, T2DM, obesity, TGF-β

## Abstract

The aim of the present study was to evaluate the involvement of CD52 in adipocytes as well as to explore its effect on type 2 diabetes mellitus (T2DM), and to improve our understanding of the potential molecular events of obesity with type 2 diabetes. Global changes in the CD52 expression patterns were detected in adipocytes and preadipocytes derived from obese and lean individuals. In particular, CD52 was identified as significantly differentially upregulated and was analyzed, both *in vitro* and *in vivo*, using various approaches. *In vitro* experiments, CD52 was a significantly up-regulated mRNA in mature adipocytes and preadipocytes. In addition, CD52 gradually increased with the differentiation of preadipocytes. *In vivo* experiments, the expression of CD52 in high-fat diet (HFD) -fed mice tended to be higher than that in regular diet (RD) -fed mice. Further analysis showed that CD52 expression was positively correlated with Smad3 and TGF-β in mice, and the downregulation of CD52 was accompanied by increased glucose tolerance and insulin sensitivity. Moreover, a comparison of CD4+CD52^high^ T cells and CD4+CD52^low^ T cells showed that many T2DM-related genes were aberrantly expressed. Overall, CD52 may functioned as an important potential target for obesity with T2DM via TGF-β/Smad3 axis.

## INTRODUCTION

Obesity is a state of excessive fat accumulation in the adipose tissue and is closely associated with many metabolic diseases such as dyslipidemia, insulin resistance, and T2DM [1, 2]. Interestingly, not all obese individuals are diagnosed with T2DM [3]. Most gene expression profiling studies have examined differences in adipose tissue gene expression between obese and lean individuals and found that obesity is accompanied by the upregulation of proinflammatory genes [4, 5]. However, these studies do not indicate that genes associated with obesity are necessarily associated with T2DM. In fact, there are some genes associated with both obesity and T2DM among obese individuals that increase the risk of T2DM.

Adipocytes are the main constituent of adipose tissue and are considered an important bridge that link obesity and T2DM due to their strong secretory function [6, 7]. In adipocytes, many cytokines are synthesized, which are related to insulin-mediated processes, including lipid metabolism and glucose homeostasis [8]. An increase in these cytokines can impair insulin signaling in the adipocyte, leading to a decrease in insulin-mediated glucose uptake and lipid accumulation, and increase ectopic lipid accumulation [9], which eventually exacerbates insulin resistance and can even lead to T2DM [10]. CD52 is a low-molecular weight glycoprotein consisting of 12 amino acids, with a glycosylphosphatidylinositol (GPI) anchored at its C-terminus [11], found in abundance on a variety of lymphoid cells, especially B and T cells, and is expressed at very high density [12]. Because the GPI anchor is cleavable by phospholipases [13], CD52 can be detached from the cell surface and become soluble CD52 [14]. Although the function of soluble CD52 is uncertain, but it seems that CD52 may be involved in migration and activation of T-cells [15], leukemia [16] and autoimmune diseases [14]. Esther’s study not only showed that CD52^high^ cells may protect humans and mice from autoimmune disease, but also indicates that transfer of lymphocyte populations depleted of CD52^high^ cells resulted in a substantially accelerated onset of Type 1 diabetes [14]. However, the roles of CD52 in T2DM is unclear. Interestingly, genomics studies indicate that CD52 is up-regulated in individuals with a phlegm-dampness constitution, and they have a much higher risk of obesity, metabolic syndrome, hypertension, and diabetes [17]. However, phlegm-dampness constitution is a diagnosis given by traditional Chinese medicine to disease and does not accurately define obesity and T2DM. In addition, while the genome sequencing results come from blood, the expression level of CD52 in adipose tissue or adipocytes is unknown.

In addition to adipocytes in adipose tissue, the remaining cellular components are preadipocytes at different stages and various immune cells such as macrophages, neutrophils, lymphocytes, and T cells, which also play a major role in obesity and diabetes [18]. CD4+CD52 T cells are components of a specialized population of Tregs with high expression of glycoprotein CD52 on the cell surface [14]. It has been reported that the release of CD52 from CD4+CD52^high^ T cells can inhibit the activation of CD4+CD52^low^ T cells, which induces these cells in a quiescent state [14, 19]. Because CD52 on the cell surface is down-regulated as resting CD4+ T lymphocytes are activated and is up-regulated after the induction of cell quiescence [20], the final result of CD52 inhibiting T cell activation is the conversion of CD4+ CD52^low^ T cells into CD4+ CD52^high^ T cells. For this reason, we suspect that there is a huge difference in gene expression between CD4+ CD52^low^ T cells and CD4+ CD52^high^ T cells, and this difference is likely to be related to insulin resistance and T2DM.

In this study, we analyzed the expression profile of mature adipocytes from lean individuals, non-diabetic obese individuals, and obese individuals with T2DM. We found that the expression level of CD52 in mature adipocytes from obese individuals with T2DM was much higher than that of non-diabetic obese individuals, and the latter was higher when compared to lean individuals. In addition, CD52 in the preadipocytes of obese patients was also significantly higher than that in lean subjects, and with the differentiation of adipocytes, the CD52 expression level was increased. Further analysis revealed that the expression of CD52 is regulated by the TGF-β/Smad3 signaling pathway, and CD52 may promote the development of T2DM by inhibiting the activation of CD52^low^ T cells.

## RESULTS

### Overexpression of CD52 in mature adipocytes of obese or diabetic patients

**Figure 1:**
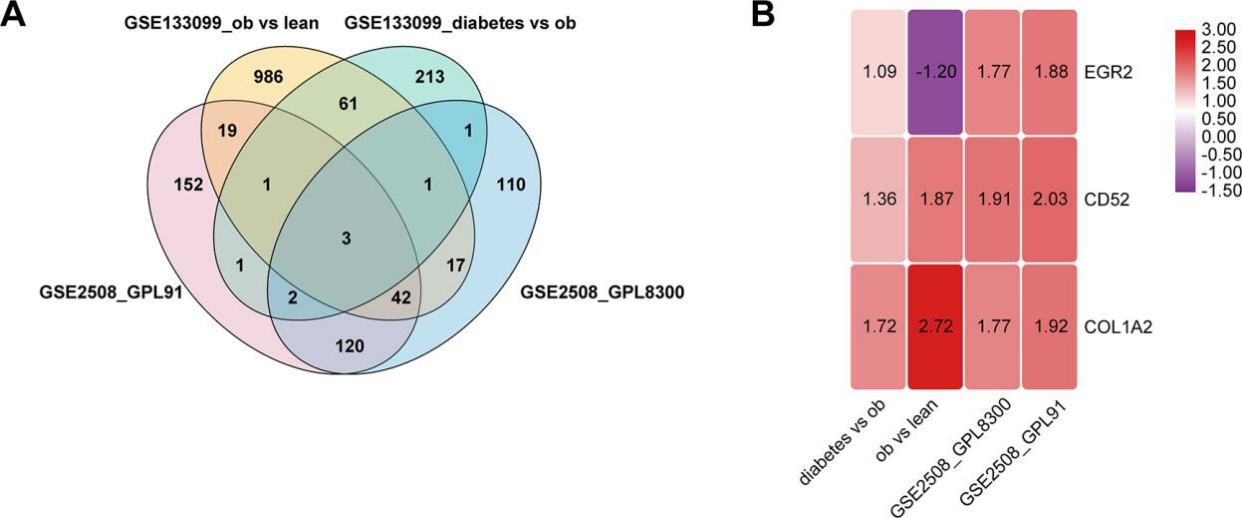
**Identification of differentially expressed genes in mature adipocytes.** (**A**) Venn diagram of the differentially expressed genes in 4 pairs group. (**B**) There are three genes that are significantly differentially expressed in all four groups, and the number in the rectangle represents logFC.

Using public microarray datasets, we studied the expression profiles of mRNA in mature adipocytes. In non-diabetic individuals, we identified 1131, 341, and 296 significantly differentially expressed mRNAs between obese and lean subjects in GSE133099, GSE2508 (GPL8300), and GSE2508 (GPL91), respectively. In obese individuals (GSE133099), we identified 283 significantly differentially expressed mRNAs between diabetic and non-diabetic individuals. In the four pairs, a total of 3 mRNAs were both significantly differentially expressed (Figure 1A). Of these, CD52 and COL1A2 were the most up-regulated mRNAs (Figure 1B).

### Time-course of CD52 changes in adipocytes during differentiation

**Figure 2:**
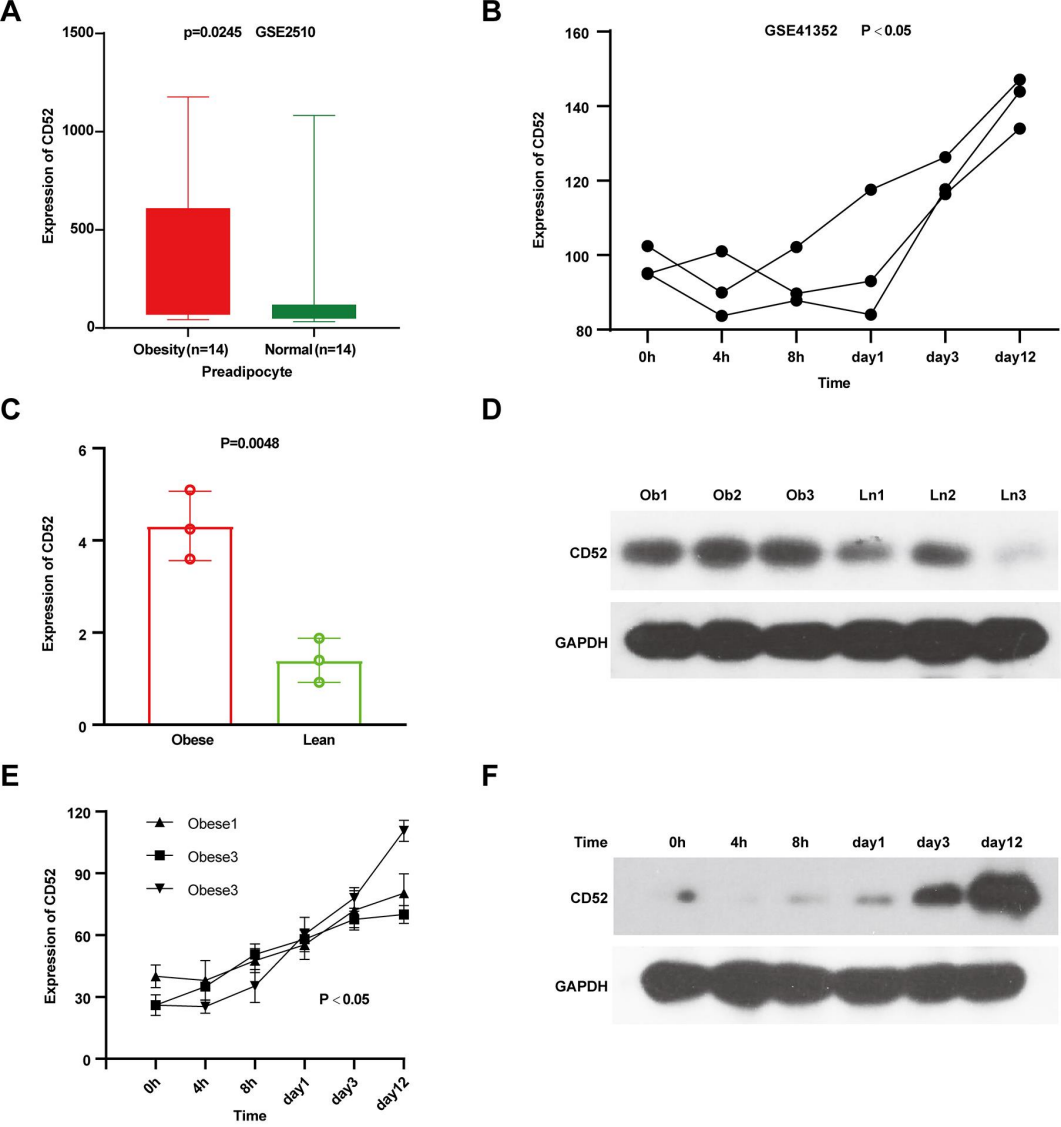
**Characterization of CD52 in preadipocytes and the process of adipocyte differentiation.** (**A**) The expression levels of CD52 in preadipocyte by microarray analysis. (**B**) The expression levels of CD52 during preadipocytes differentiation by microarray analysis. (**C**, **D**) The expression levels of CD52 in preadipocyte between three obese people and three lean people by Real-time RT-PCR and western blot analysis. (**E**, **F**) The expression levels of CD52 during preadipocytes differentiation by Real-time RT-PCR and western blot analysis.

To assess CD52 expression changes during adipocyte differentiation, we first analyzed whether CD52 was up-regulated in the preadipocytes of obese individuals. After comparing the preadipocyte samples, it was determined that CD52 was highly expressed in obese individuals (Figure 2A, P = 0.0245, 14 obese vs 14 lean, GSE2510). Additionally, CD52 mRNA expression was also consistently up-regulated during the development of adipocyte differentiation over a 12-day period (Figure 2B, GSE41352). To further evaluate the expression level of CD52 in the preadipocytes and its change during differentiation, qRT-PCR and western blot experiments were carried out. The results showed that CD52 expression levels were found to be significantly up-regulated in the preadipocytes of 3 obese patients without diabetes relative to 3 lean controls (Figure 2C, 2D). Also, there was a significant up-regulated in adipocytes during differentiation (Figure 2E, 2F).

### Associations between CD52 expression and the TGF- β/Smad3 signaling pathway

**Figure 3:**
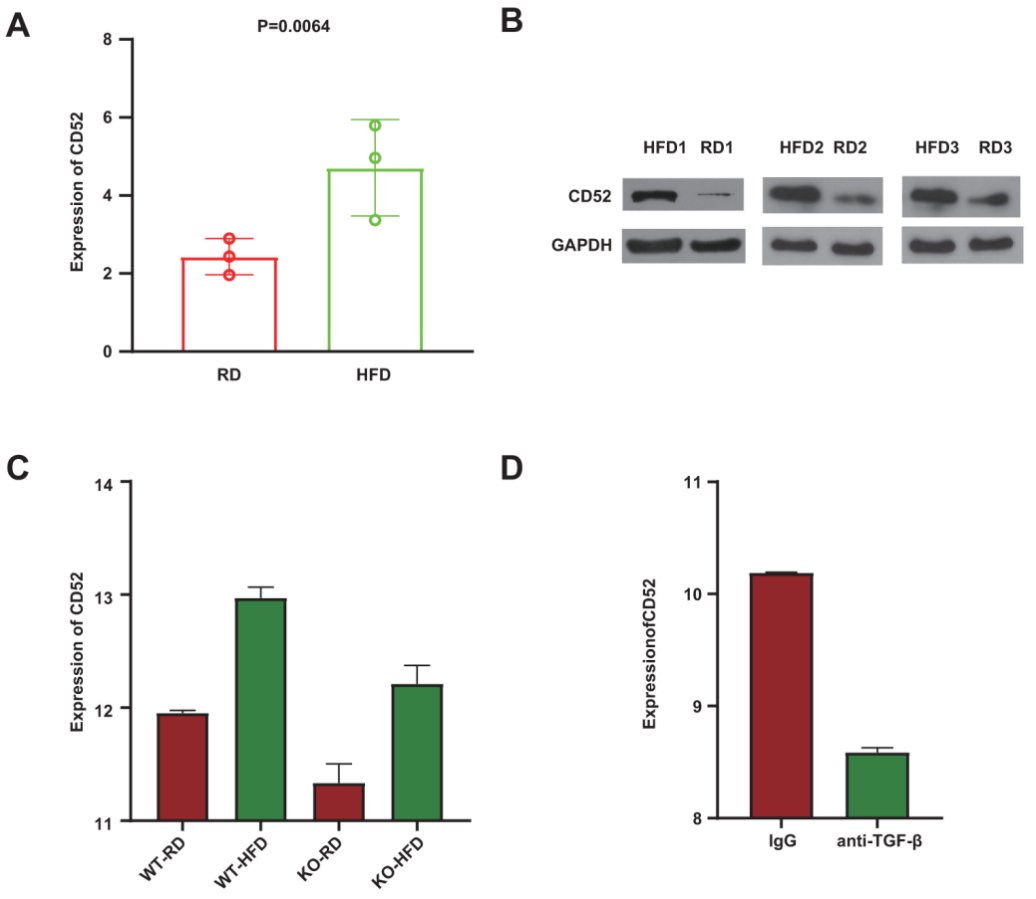
**Anti-TGF-β/Smad3−/− mediated CD52 downregulation counteracts HFD-induced obesity and insulin resistance.** (**A**, **B**) The expression levels of CD52 in preadipocytes from RD-fed or HFD-fed mice by Real-time RT-PCR and western blot analysis. (**C**) The expression levels of CD52 in WAT from RD-fed or HFD-fed KO mice and WT mice, and the expression levels of CD52 in WAT from HFD-fed KO mice and HFD-fed WT mice by microarray analysis. (**D**) The expression levels of CD52 in WAT from HFD-fed mice treated with anti-TGF-β antibody (1D11) and HFD-fed mice treated with control IgG by microarray analysis.

To further explore CD52 in preadipocytes, CD52 expression was examined from preadipocytes of 3 mice fed regular diet (RD) and 3 mice fed high-fat diet (HFD) using qRT-PCR and western blot. And significantly increased expression of CD52 was observed in preadipocytes of mice fed high-fat diet (HFD) (Figure 3A, 3B). Previous studies have shown that TGF-β/Smad3 plays an important role in promoting diet-induced diabetes [21, 22]. To further investigate whether CD52 is the transcriptional regulatory target of the TGF-β/Smad3 signaling pathway, a microarray of white adipose tissue (WAT) isolated from Smad3+/+ mice (WT) and Smad3−/− mice (KO) fed either a regular diet (RD) or a high-fat diet (HFD) was analyzed (based data in GSE28598). The results showed that regardless of whether the sample originated from the WT group or the KO group, the expression of CD52 in HFD-fed mice tended to be higher than that in RD-fed mice (Figure 3C, P = 4.78E-07, WT-HFD vs WT-RD; P = 2.57E-04, KO-HFD vs KO-RD, GSE28598). Moreover, in the HFD group, the expression of CD52 in WT mice tended to be higher than that in KO mice (Figure 3C, P = 2.65E-05, WT-HFD vs KO-HFD, GSE28598). In addition, in the microarray analyses of WAT from diet-induced obese (DIO) mice, we observed significantly increased expression of CD52 in mice treated with IgG, as compared to mice treated with anti-TGF-β antibody (1D11) (Figure 3D, P = 1.69E-06, GSE28598). The studies thus far indicated a beneficial effect of suppressing TGF-β/Smad3 signals on glucose tolerance. To examine these findings, we also observed the expression of typical genes that are beneficial to improving insulin resistance, i.e., PGC-1α [23], PGC-1β [24], DIO2 [25], UCP1 [26], and PRARγ [27]. Our results showed increased mRNA expression of these genes in the group with low expression of CD52 (Supplementary Figure 1, GSE28598).

### Comparison of CD4+CD52^high^ T cells and CD4+CD52^low^ T cells

**Figure 4:**
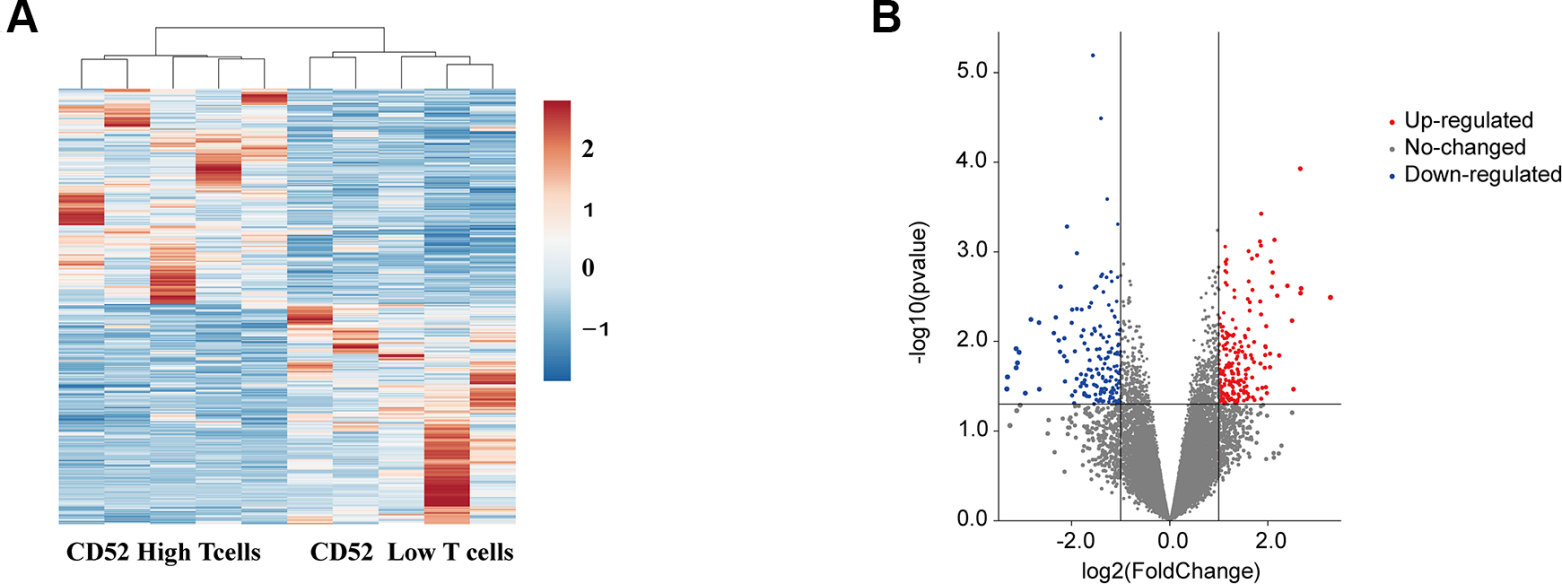
**Identification of differentially expressed mRNAs in different CD4+ T cells.** (**A**) Clustered heat map of the differentially expressed mRNAs between CD52^high^ T cells CD52 ^low^ T cells. up-regulated mRNAs are shown in red, and down-regulated mRNAs are shown in blue. (**B**) Volcano plots comparing the expression of mRNAs in between CD52^high^ T cells CD52 ^low^ T cells. The red dots represent the significantly up-regulated differentially expressed mRNAs (fold-change ≥ 2 and P < 0.05), the blue dots represent the significantly down-regulated differentially expressed mRNAs (fold-change ≤ -2 and P < 0.05).

Considering the elevated levels of CD52 expression in the adipocytes of obese and obese T2DM patients, it is likely that this may lead to an increase in the ratio of CD4+CD52^high^ T cells to CD4+CD52^low^ T cells, thereby promoting the development of diabetes. To further confirm whether CD52 is involved in the pathogenesis of diabetes, we performed microarray analyses of CD4+CD52^high^ T cells and CD4+CD52^low^ T cells based on GSE94815. Compared to CD4+CD52^low^ T cells, 222 genes were up-regulated and 177 genes were down-regulated (P <0.05 and FC ≥ 2 or FC ≤ 1/2,). These aberrant genes are presented as an expression of heatmap and volcano plot (Figure 4A, 4B).

### WGCNA

**Figure 5:**
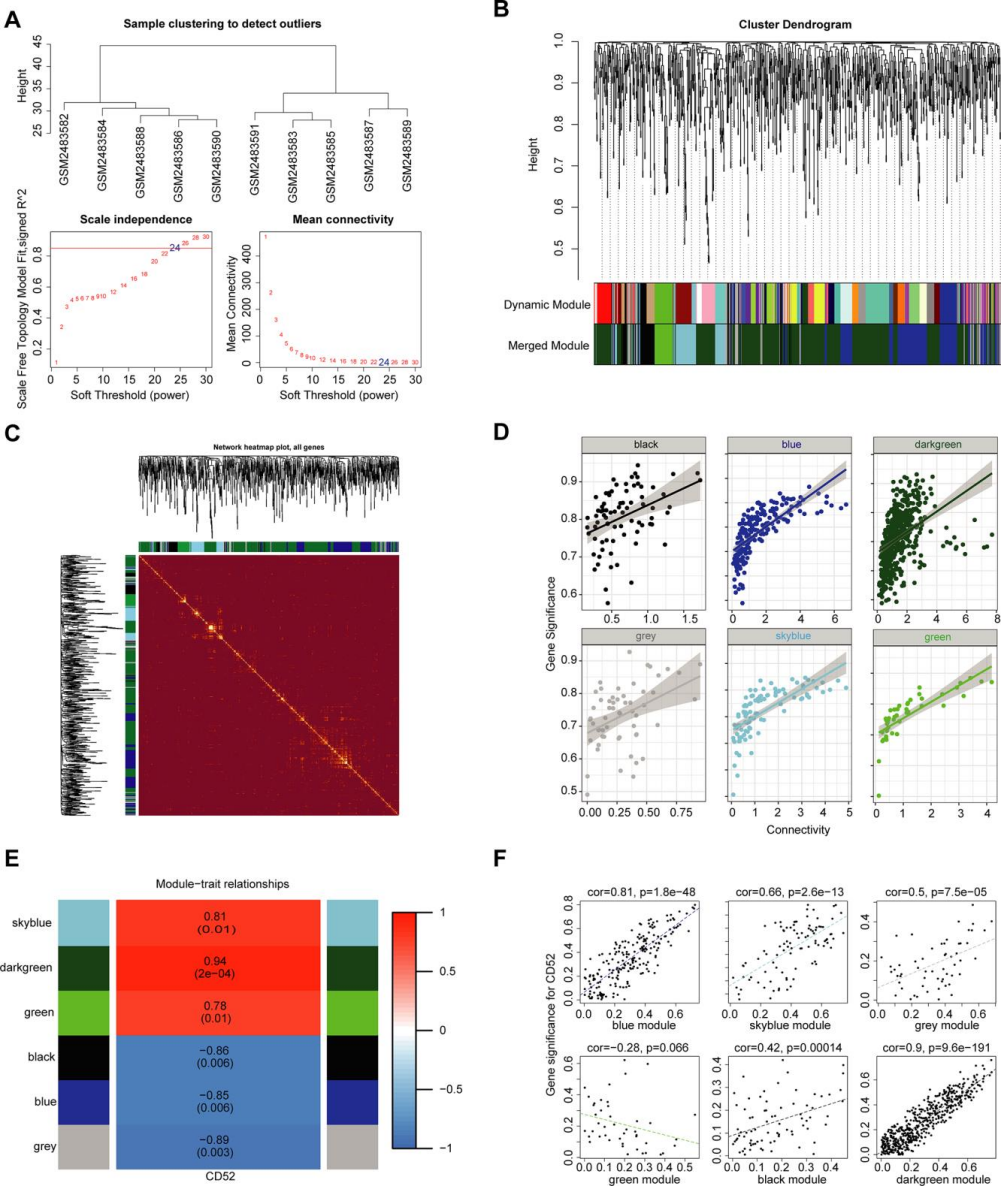
**WGCNA.** (**A**) Analysis of the scale-free topology model fit index for various soft-thresholding powers (β) and the mean connectivity for various soft-thresholding powers. Overall, 24 was the best fitting power value. (**B**) Dendrogram of the gene modules based on a dissimilarity measure. The branches of the cluster dendrogram correspond to the different gene modules. Each piece of the leaves on the cluster dendrogram corresponds to a gene. (**C**) TOM Diagram of Gene clustering and Module relationship in each Module of WGCNA. (**D**) The relationship between intra-module connectivity and gene significance in each module of WGCNA. (**E**) Module-trait relationships. Heatmap of the correlation between module eigengenes and expression of CD52. (**F**) The relationship between each module and clinical features. The horizontal axis represents the correlation coefficient between gene expression and module, and the vertical axis represents the correlation coefficient between gene expression and phenotype.

We used the expression profiles of 1002 significantly differently expressed mRNAs (P < 0.05) in GSE94815 to construct a coexpression network with the WGCNA software package in R software. In the coexpression network analysis, the β values were 24 (Figure 5A). The results of screening module and gene clustering are shown in Figure 5B. The clustering relationship between the WGCNA module and the module and the correlation coefficient distribution between the module and the gene expression within the module are shown in Supplementary Figure 2. Figure 5C shows the TOM diagram of gene clustering and module relationship in each module of WGCNA. The relationship between intra-module connectivity and gene significance in each module of WGCNA is shown in Figure 5D, and it is obvious that the darkgreen module is the most significant. Ultimately, we obtained 6 modules in the coexpression network of mRNAs (Figure 5E). Moreover, we calculated and plotted the relationship between each module and clinical features. However, as shown in Figure 5F, there is a significant positive correlation between the darkgreen module and CD52 characteristics (module-feature weighted correlation = 0.90, P = 9.6E^-191^).

### Functional enrichment analysis

**Figure 6:**
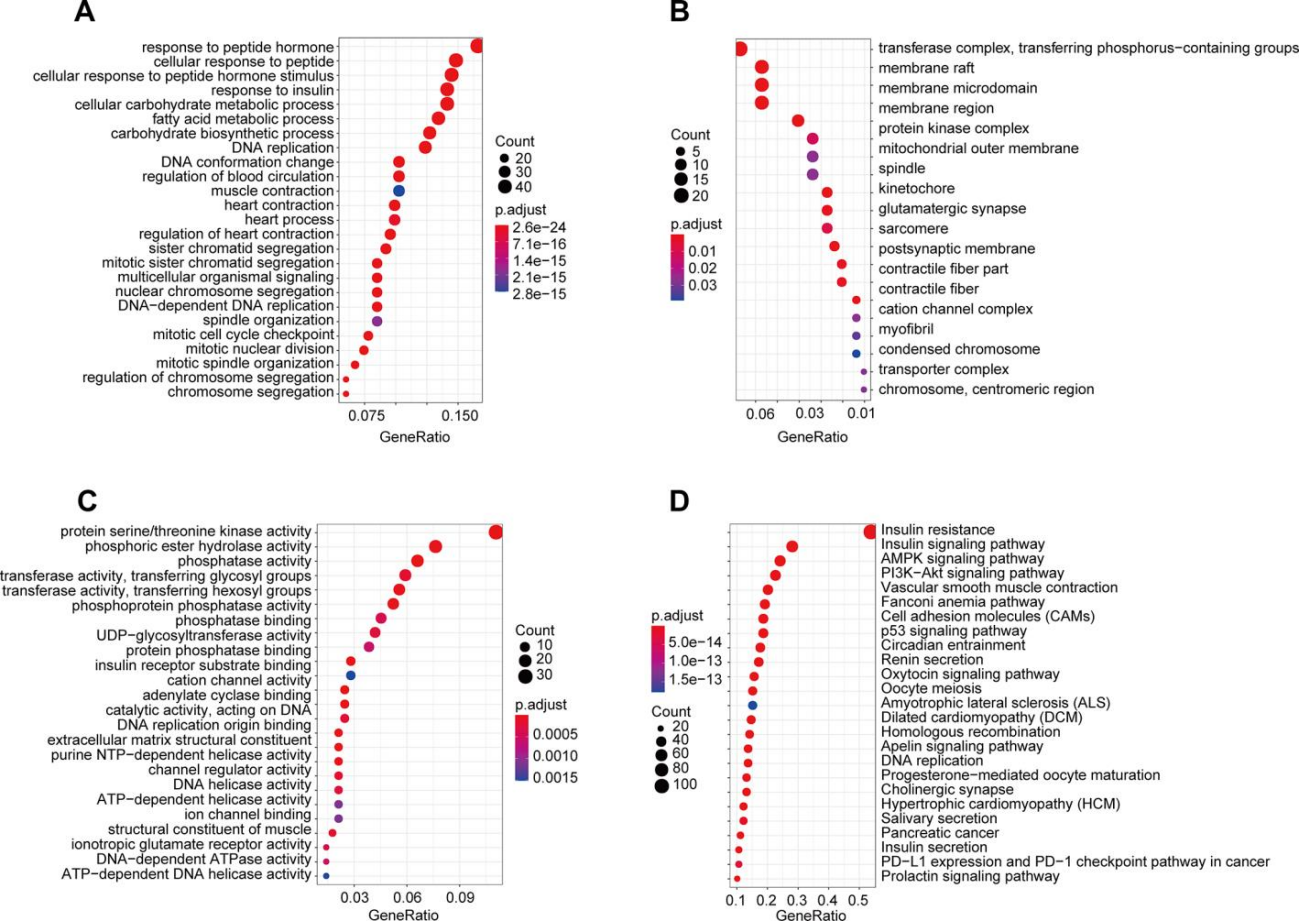
**GO and KEGG pathway enrichment of genes in the darkgreen module.** (**A**) Biological process; (**B**) cellular component; (**C**) molecular function; (**D**) Kyoto Encyclopedia of Genes and Genomes (KEGG) pathways.

Differentially expressed gene functions and pathways, acting as important functional units of gene groups, play key biological roles in the development and progression processes of many diseases. Thus, we analyzed pathway enrichment and functions using the 525 genes in the darkgreen module in WGCNA. The results indicated that the enriched biological processes mainly involved response to insulin, response to peptide hormone, glucose metabolic process, cellular response to insulin stimulus and so on (Figure 6A). The cell components that were correlated with the resulting terms included transferase complex, transferring phosphorus−containing groups, membrane raft, membrane microdomain, membrane region and so on (Figure 6B). The results also showed that the molecular functions were related to protein serine/threonine kinase activity, phosphoric ester hydrolase activity, phosphatase activity, transferase activity, transferring glycosyl groups, phosphoprotein phosphatase activity and so on (Figure 6C). KEGG pathway analysis showed that these genes were mainly enriched in Insulin resistance, Insulin signaling pathway, AMPK signaling pathway, and PI3K−Akt signaling pathway (Figure 6D).

These functions and pathways were consistent with our known understanding regarding T2DM and might further explain the involvement between CD52 and the pathogenesis of T2DM.

### PPI network and hub gene

**Figure 7:**
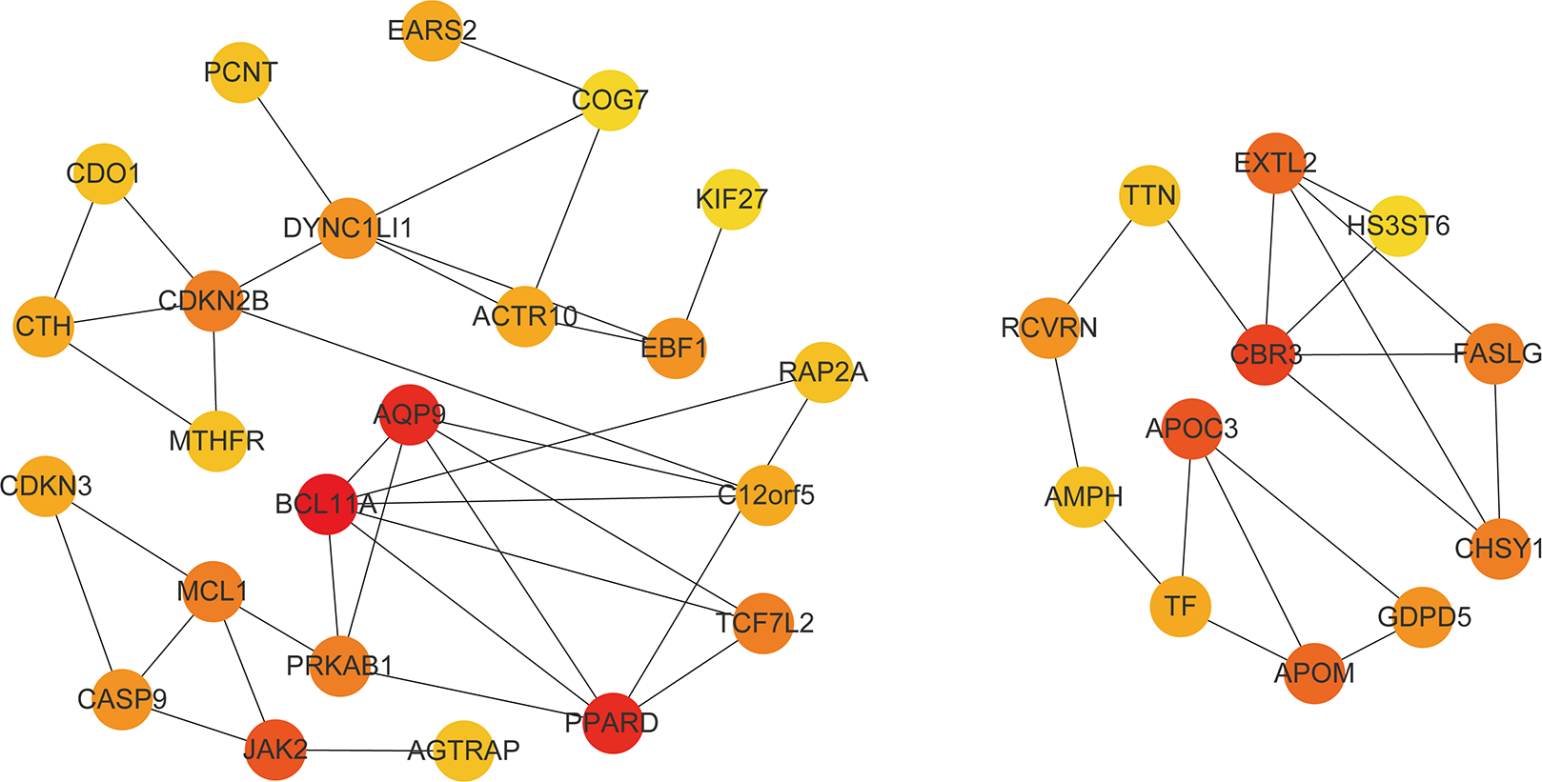
**Hub gene network.** The figure contains the first 35 gene with the highest network connectivity. The darker the color, the higher the connection.

After PPI network analysis of 525 genes in the darkgreen module of WGCNA, we obtained a network diagram containing all the interacting proteins (Supplementary Figure 3). In the analysis of hub gene network, we extracted the first 35 gene with the highest network connectivity (Figure 7). Interestingly, 20 of these gene are closely related to the occurrence and development of diabetes. Among the 20 molecules, the up-regulated (P <0.05 and FC ≥ 2) genes included: (1) genes involved in insulin resistance such as APOC3 [28], PPARD [29], CASP9 [30], and CBR3 [31]; (2) risk loci for type 2 diabetes such as MTHFR [32] and CDKN2B [33]; (3) genes correlated with complications of T2DM; for instance, casp-9, which mediates high-glucose-induced diabetic neuropathies [34]; EBF1, which is a cardiovascular and metabolic risk gene [35]; and APOM, which is associated with lipid disturbances and rheumatoid arthritis [36]; (4) BCL11A, which is a candidate regulator of pancreatic endocrine cells, downregulates target genes Ins2, glucagon, and Ppy [37]. The down-regulated genes (P <0.05 and FC ≤ 0.5) included: (1) genes modulating the lipolytic program and promoting brown adipose tissue function, such as JAK2 [38]; (2) genes downregulating insulin resistance, such as PCNT [39], RAP2A [40], AQP9 [41], MCL1 [42], AGTRAP [43], TF [44] and FASLG [45]; and (3) TCF7L2, which plays an important role in glucose homeostasis [46]; (4)Human GDPD5 restores insulin expression in Gdpd5a-depleted zebrafish embryos [47]. These up-regulated genes and down-regulated genes are respectively presented in Supplementary Tables 1, 2.

## DISCUSSION

Obesity is significantly closely associated with T2DM, which is characterized by a decreased response to insulin signaling in several types of peripheral tissues, including adipose, liver, and muscle [48]. However, not all obese patients have T2DM [49] and many non-obese patients do [50]. This suggests that there may be some genes that are related to both obesity and T2DM. These genes probably cause type 2 diabetes on the basis of obesity. In order to identify these genes, we performed microarray analyses of mature adipocytes between obese and lean individuals, and mature adipocytes between obese patients and obese T2DM patients. Finally, we selected the intersection of the two differentially expressed genes that were most relevant to obesity and diabetes. After focusing on only the most profoundly up-regulated and the most profoundly down-regulated genes known to affect these two conditions, CD52 was identified, and this has never been reported in adipocytes.

Cluster of differentiation (CD) Ags are cell surface molecules expressed on leukocytes and other cells involved in the immune system. They are commonly used as cell markers, allowing for the identification and isolation of leukocyte populations and subsets [51]. CD52 is a newly discovered leukocyte differentiation antigen. It was first found in humans as expressed on the surface of lymphocytes, monocytes, and eosinophils [52]. Previous studies have shown that CD52 in blood is correlated with T2DM and obesity [17]. However, to our knowledge, there are no reports on CD52 involved in obesity and T2DM in adipocytes. Our study found that the level of expression of CD52 in adipocytes from obese patients was higher than in adipocytes from lean people. Obese patients with T2DM also showed a higher level of CD52 than obese non-diabetic patients. All of these results further indicate that CD52 has important value for the study of obesity combined with T2DM.

Preadipocytes and mature adipocytes are the two main populations studied in the adipocyte differentiation process. It has been reported that preadipocytes and mature adipocytes assume different functions during the differentiation process [53]. Various investigators have also shown that preadipocytes and mature adipocytes have different gene transcription levels [54]. Our data clearly show that CD52 in preadipocytes differed significantly between obese patients and lean people, and the level of expression of CD52 gradually increased with the differentiation of preadipocytes. During the differentiation of preadipocytes, the lipolytic capacity of adipocytes was significantly reduced, while the lipid synthesis capacity increased [55, 56]. Adiposome-derived GPI proteins within the adipocytes were found to mediate the inhibition of lipolysis [57]. Because it is a GPI protein, the elevation of CD52 at the terminal differentiation stage is also likely related to lipolysis. If adipocytes are unable to carry excess energy, the calories are stored in the liver, muscles, and blood, and insulin resistance and T2DM ultimately result [55].

Transforming growth factor beta (TGF-β) is a multifunctional growth factor that plays important roles in cell growth and differentiation, extracellular matrix deposition, cell adhesion, and immunomodulation [58]. It has been reported that appropriate TGF-β suppression may have therapeutic value for diabetic patients who are also obese [22]. Furthermore, some studies also showed that TGF-β can regulate cluster of differentiation (CD) expression [59]. TGF-β exerts its biological functions mainly through its downstream signaling molecules, such as Smads [60]. For this purpose, we examined the expression of CD52 in the adipose tissue of genetically obese mice (ob/ob mice), TGF-β-deficient mice, and Smad3 knockout (KO) mice based on GSE28598. The results show that Smad3 deletion and anti-TGF-βantibody each reduced CD52 expression. In addition, as previously reported, some genes beneficial to glucose tolerance were up-regulated in the group with low expression of CD52, suggesting that high expression of CD52 is associated with insulin resistance. Smad3-/-mice (KO) exhibited more insulin sensitivity than Smad3+/+ mice (WT), as evidenced by elevated glucose infusion rate and increased whole-body glucose uptake, during a hyperinsulinemic-euglycemic clamp experiment [22]. When challenged with a high-fat diet (HFD), Smad3-/-mice (KO) exhibited enhanced glucose tolerance and insulin sensitivity, leading to lower fasting blood glucose and insulin levels [22]. Compared to animals treated with the isotype control 13C4 antibody (IgG), mice treated with anti-TGF-β (α-TGF-β) antibody had significantly lower fasting blood glucose and fasting insulin levels [22]. These results offer insight into the role of CD52 in adipose tissue biology, specifically with regard to a strong potential for translation of these observations for the treatment of obesity and diabetes.

CD52 on the cell surface and soluble CD52 appear to have different mechanisms [15]. Cross-linkage of CD52 molecules by an as-yet unidentified endogenous ligand that is mimicked by a bivalent anti-CD52 antibody results in the expansion of CD52-expressing T cells [15]. Soluble CD52 released from the cell surface of CD4+CD52^high^ T cells results in inhibition of the proliferation of CD4+CD52^low^ T cells by preventing the activation of these cells [14, 15]. Thus, elevation of soluble CD52 can cause an imbalance in the ratio of CD4+ CD52^low^ T cells to CD4+ CD52^high^ T cells, and it ultimately results in an increase in the number of CD4+ CD52^high^ T cells. After comparing CD4+CD52^high^ T cells to CD4+CD52^low^ T cells, we found many aberrantly expressed genes, some of which were up-regulated and found to play a role in promoting the development of T2DM, and some of which were down-regulated and act to protect against T2DM. The consistency in the role CD52 played with these aberrantly expressed genes in T2DM could also be seen in the functions and pathway enrichment. These results suggest that the effect of CD52 on T cells may be an important mechanism underlying its impact on T2DM.

In summary, our study provides a potential gene target for adipocytes that promote T2DM. High expression of CD52 in adipocytes may be an adverse biomarker for obesity and T2DM. Functionally, CD52 is involved in adipocyte differentiation and the TGF-/Smad3 signaling pathway and influences CD4+CD52^low^ T cells. Taken together, our results complement the role of CD52 in obesity and metabolic diseases and offers a unique opportunity for the treatment of T2DM.

## MATERIALS AND METHODS

### Clinical samples

Adipose tissue samples were prospectively collected from 3 patients undergoing laparoscopic hernia repair [in lean (Ln) volunteers] and 3 patients undergoing bariatric surgery [in obese (Ob) subjects] at the Third People’s Hospital of Chengdu, China between June 2018 and July 2019. The specimens were frozen with liquid nitrogen immediately after removal and transferred to the −80° C refrigerator. According to the China National Nutrition and Health Survey (CNNHS) data, a BMI of ≥28 kg/m^2^ in Chinese adults suggests obesity [61]. This study was approved by the Institutional Ethics Review Board of the Third People’s Hospital of Chengdu (record #: 2018S75; Chengdu, Sichuan, China), and was conducted in accordance with the Chinese ethical guidelines for human genome/gene research.

### Preadipocyte isolation

Preadipocytes from visceral adipose tissue (VAT) were isolated and cultured following standard protocols [62]. In brief, VAT was digested with collagenase to obtain stromal cells. Stromal cells were separated from mature adipocytes by centrifugation and then incubated in erythrocyte lysis buffer for 10 min at room temperature to eliminate red blood cells. The remaining debris was removed by filtering the cell suspension through a 70-μm nylon filter and centrifuging the filtrate. Pelleted preadipocytes were plated in basal medium consisting of DMEM/F-12 (Gibco, Carlsbad, CA) supplemented with 10% fetal calf serum (FCS) and incubated for 16–18 h. After incubation, attached cells were washed thoroughly with warm PBS, removed from plates with trypsin, resuspended, and counted.

### Animals [63]

C57BL/6J mice (9-weeks-old) were kept in a pathogen-free facility and maintained under a 12 h light–dark cycle at 22° C. 3 mice were fed ad libitum with a high-fat diet (HFD; TD88137 Harlan Teklad) and 3 mice were fed ad libitum with a regular diet (RD). VAT were excised and isolation of preadipocytes [63]. Animal care and experimental procedures were approved by the Ethics Committee in Animal Experimentation of West China Hospital, Sichuan University, Chengdu, China (record #: 2019014A).

In the GEO database GSE28598 data set, Smad3+/+ mice (WT) and Smad3−/− mice (KO) were fed with a regular diet (RD) or 55% high fat diet (HFD) for 8 weeks. Diet-induced obese (DIO) mice were intraperitoneally injected with 1.5 mg/kg body weight of control 13C4 antibody (IgG) or anti-TGF-β antibody (1D11) three times a week for 8 weeks [22].

### Cell culture and differentiation

Preadipocytes were cultured in Dulbecco’s modified Eagle’s medium (DMEM)/Nutrient Mix F12 (Gibco) containing 8 mg/l biotin, 4 mg/l pantothenate, 0.1 mg/mg streptomycin and 100 U/ml penicillin (OF medium) supplemented with 10% FBS in a humidified 95%air/5%CO2 incubator. The cells were seeded into culture medium flasks or plates, which were coated with a solution of 10 microL/ml fibronectin and 0.05% gelatine in phosphate-buffered saline. Confluent cells were cultured in serum-free OF medium for 2 days followed by stimulation to differentiate with OF media supplemented with 0.01 mg/ml human transferrin, 200 nM T3, 100 nM cortisol, 20 nM insulin, 500 microM IBMX and 100 nM rosiglitazone (Cayman Chemicals). After day 4, the differentiating cells were kept in OF media supplemented with 0.01 mg/ml human transferrin, 100 nM cortisol and 20 nM insulin. Preadipocytes differentiate within 10–12 days as determined by microscopic analysis. RNA samples were collected at 0, 4, 8 and 12 h and on days 1, 3 and 12 of differentiation [63]. The expression levels of C/EBP-α and PPAR-γ, which are biomarkers of preadipocyte differentiation [62].

### Microarray and sequencing data analyses

Several previously published datasets were used for gene expression profiles, including GSE133099, GSE2510, GSE2508, GSE41352, GSE28598, and GSE94815, all of which can be obtained from the NCBI-GEO (https://www.ncbi.nlm.nih.gov/gds/) database. Microarray expression profiles were obtained by Illumina HiSeq 2500 (Homo sapiens), Affymetrix Human Genome U133A, Illumina HumanHT-12 V3.0 expression beadchip, Affymetrix Mouse Genome 430 2.0, and Agilent-039494 SurePrint G3 Human GE v2 8x60K Microarray 039381. All of the design, quality control, and data normalization for all experiments was in accordance with the standard protocols.

### Western blot analysis

Proteins were extracted from cultured cells or adipose tissue using RIPA lysis buffer. The protein concentration was determined with a bicinchoninic acid protein assay kit (Sigma). Proteins were separated by 12% SDS-PAGE and transferred to PVDF membranes. After blocking for 1 h, the membranes were incubated with primary antibody at 4C overnight. Membranes were incubated with the appropriate HRP-conjugated secondary antibody at room temperature for 2 h. The immunoreactive bands were visualized using ECL and normalized to GAPDH (the internal control).

### Real-time RT-PCR

Total RNAs were reverse transcribed to complementary cDNA using Transcriptor First Strand cDNA Synthesis Kit (Roche, Penzberg, Germany), following the manufacturer’s instructions. Quantitative gene expression was measured by real-time RT-PCR using the FastStart Essential DNA Green Master Mmix (Roche, Penzberg, Germany) on a Roche LightCycler 480 (Roche, Penzberg, Germany). RNA expression was normalized to GAPDH expression. All quantitative PCRs were conducted in triplicate. The sequence of primers is shown in Supplementary Table 3.

### Microarray analysis

Differentially expressed RNAs were identified by the edgeR [64] package in R software. Significantly expressed RNAs were identified by setting the adjusted *P* value to < 0.05 and the |log_2_FC (fold change) | > 1(|log_2_FC > 1| and adjusted *FDR* < 0.05). GO and KEGG analyses were realized through the org.Hs.eg.db package and clusterProfiler in R software. GO consists of three terms: biological process (BP), molecular function (MF), and cellular composition (CC). All important GO terms and KEGG pathways were filtered according to a P < 0.05 and at least two associated mRNAs.

### Construction of the weighted gene coexpression network

The WGCNA [65] package implemented in R software was used to build a gene coexpression network based on the gene expression characteristics. A scale-free plot was used to evaluate whether the network exhibited scale-free topology. The power value of the soft threshold of the adjacency matrix met the scale-free topology criterion. On this basis, we built a scale-free network and topological overlap matrix (TOM). The dynamic tree cutting method was used to generate modules with the following main parameters: deepSplit of 2 and min module size of 10. The height cut-off was set to 0.25, and if the module’s similarity was > 0.8, the modules were merged. Based on Pearson’s tests, we further determined the association between module eigengenes (MEs) and external clinical information, including sample status. If the P-value was < 0.05 and the correlation coefficient was > 0.9, it was considered a significant correlation.

### Protein protein interaction (PPI) network

All the genes in the module most positively related to CD52 expression in WGCNA analysis were analyzed by PPI through STRING website (https://string-db.org/). Finally, visualization and hub network analysis are carried out with Cytoscape (version 3.7.2) [66].

### Statistical analyses [67]

All statistical analyses were performed with R (version 3.6.3) and SPSS v26.0 (SPSS Inc, Chicago, IL). Statistical significance between groups was determined using two tailed Student s t test. P value of <0.05 was considered statistically significant and all tests were two sided.

### Data availability statement

The data from TCGA and GEO datasets in this study are publicly available.

## Supplementary Material

Supplementary Figures

Supplementary Tables
