# Supplementary material for: High expression of CD52 in adipocytes: a potential therapeutic target for obesity with type 2 diabetes
Source: Aging (Albany NY). 2021 Mar 11;13(8):11043–60. doi: 10.18632/aging.202714 (PMC8109061; doi:10.18632/aging.202714)
Supplement: Supplementary Figures [file aging-13-202714-s001.pdf]

SUPPLEMENTARY FIGURES

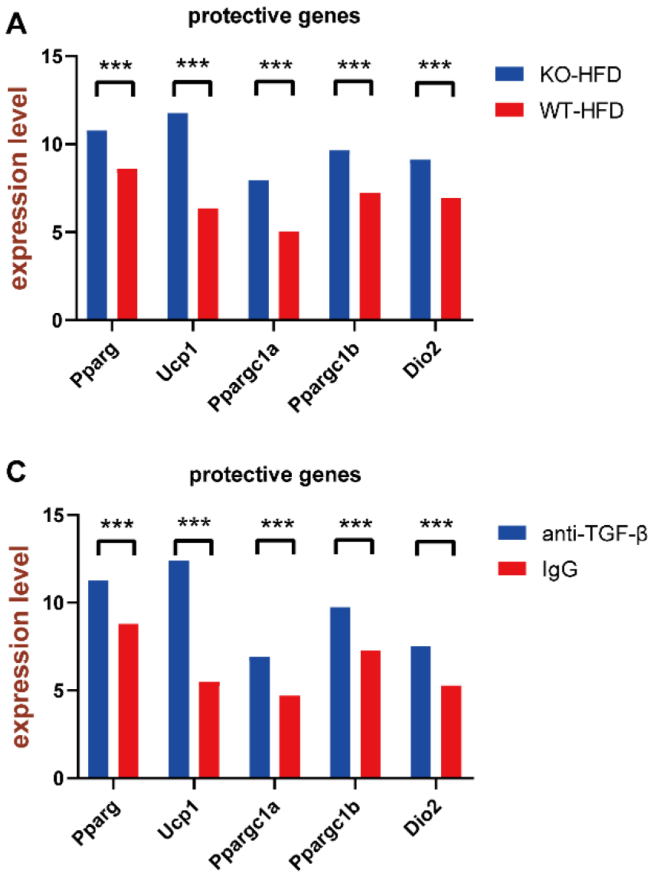

Supplementary Figure 1. The expression of typical genes that are beneficial to improving insulin resistance. (**A**) The expression of PGC-1α, PGC-1β, DIO2, UCP1, and PRARγ between KO-HFD and WT-HFD group. (**B**) The expression of PGC-1α, PGC-1β, DIO2, UCP1, and PRARγ between KO-RD and WT-RD group. (**C**) The expression of PGC-1α, PGC-1β, DIO2, UCP1, and PRARγ between anti-TCGA-β and IgG group.

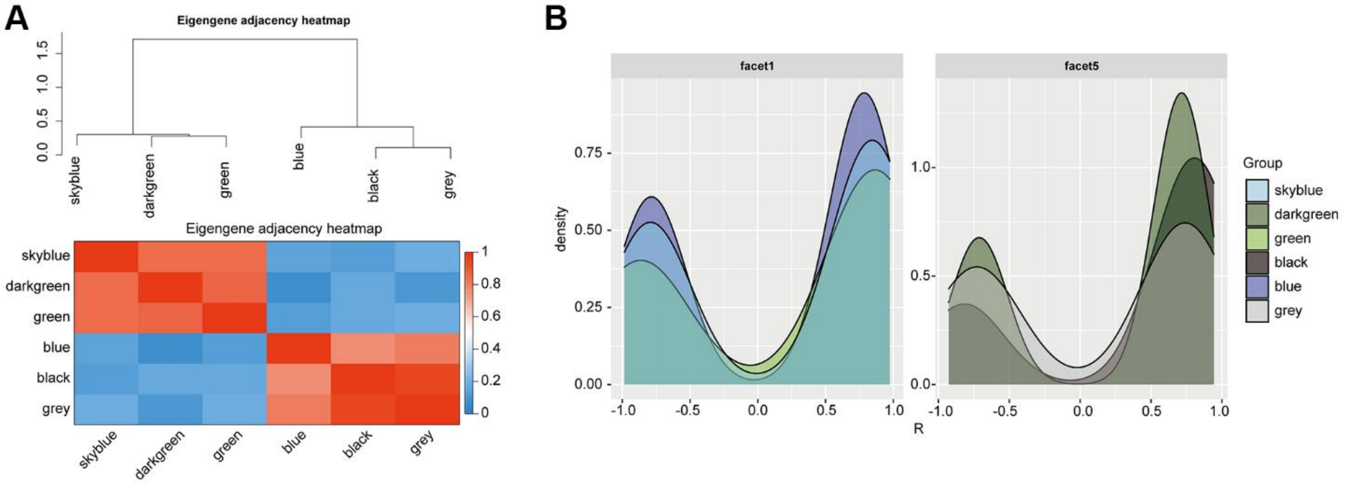

Supplementary Figure 2. The clustering relationship between the WGCNA module and the module (**A**) and the correlation coefficient distribution between the module and the gene expression within the module (**B**).

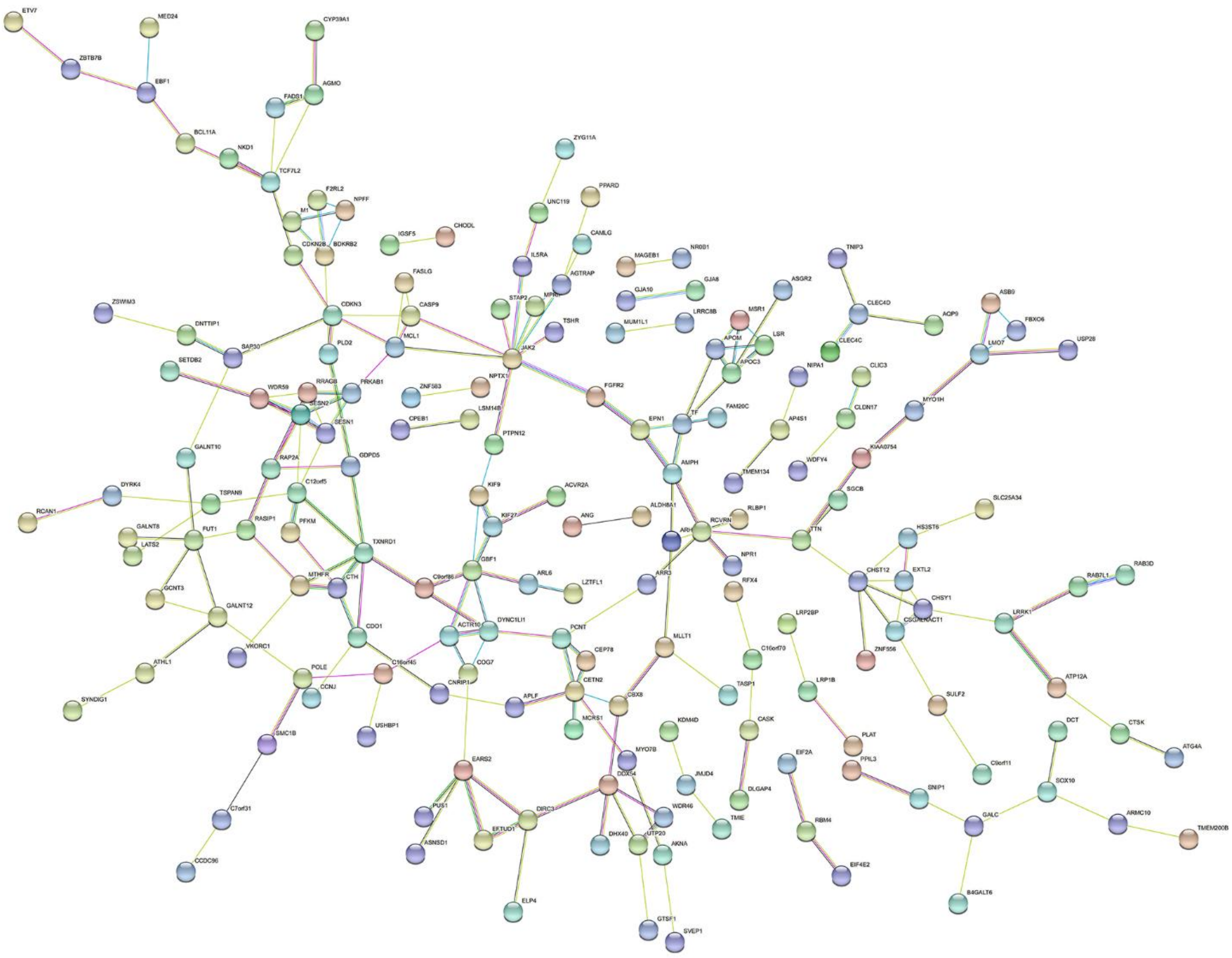

Supplementary Figure 3. PPI network.
