# Supplementary material for: High expression of CD52 in adipocytes: a potential therapeutic target for obesity with type 2 diabetes
Source: Aging (Albany NY). 2021 Mar 11;13(8):11043–60. doi: 10.18632/aging.202714 (PMC8109061; doi:10.18632/aging.202714)
Supplement: Supplementary Tables [file aging-13-202714-s002.pdf]

SUPPLEMENTARY TABLES

Supplementary Table 1. The up-regulated genes associated with T2DM.

Genes	Fold -change	P-value
**EBF1**	1.72	0.002
**BCL11A**	1.69	0.001
**CBR3**	1.62	0.021
**CDKN2B**	1.41	0.013
**APOM**	1.87	0.033
**APOC3**	1.95	0.005
**MTHFR**	1.84	0.013
**casp-9**	1.02	0.041
**PPARD**	1.34	0.022
**CASP9**	1.32	0.018

Supplementary Table 2. The down-regulated genes associated with T2DM.

Genes	Fold -change	P-value
**JAK2**	-1.29	0.016
**AQP9**	-1.64	0.001
**GDPD5**	-2.05	0.004
**FASLG**	-2.81	0.035
**TCF7L2**	-1.30	0.016
**PCNT**	-1.83	0.002
**RAP2A**	-2.03	0.027
**MCL1**	-1.79	0.003
**AGTRAP**	-1.33	0.018
**TF**	-1.75	0.001

Supplementary Table 3. Primer sequences for qRT-PCR.

Gene name	Forward primer	Reverse primer	bp
CD52	5'- CTGCCCTTACCAGAGCTGAAA-3'	5'-TCCTTTCCAGCTGTCCCTAGA-3'	64
GAPDH	5'- GAAAGCCTGCCGGTGACTAA -3'	3'- GCCCAATACGACCAAATCAGAG -5'	150

qRT-PCR, quantitative real-time polymerase chain reaction.
